# *Mycobacterium avium* complex (MAC) elimination slopes from sputum are potential treatment response biomarkers in patients with HIV and pulmonary MAC

**DOI:** 10.5588/ijtldopen.25.0313

**Published:** 2026-02-11

**Authors:** J.G. Pasipanodya, L. Nqwata, J. Schmitz, S. Srivastava, C.N. Wanjalla, R.S. Wallis, K.E. Dooley

**Affiliations:** 1 Division of Infectious Diseases, Vanderbilt University Medical Center, Nashville, TN, USA;; 2 Division of Pulmonology, Department of Internal Medicine, University of Witwatersrand, Johannesburg, South Africa;; 3 Division of Infectious Diseases, Department of Medicine, School of Medicine at University of Texas at Tyler, Tyler, Texas, USA;; 4 The Aurum Institute, Johannesburg, South Africa.

**Keywords:** non-TB mycobacteria, all-cause mortality outcomes, bacterial elimination, South Africa

## Abstract

**BACKGROUND:**

Lack of quantitative and reproducible treatment response biomarkers is slowing the development of better therapy regimens for pulmonary *Mycobacterium avium* complex (P-MAC) disease. We hypothesised that steeper bacterial elimination rate slopes (BERSs) would be associated with faster mycobacterial cure and more favourable outcomes.

**METHODS:**

We retrospectively compared serial sputum culture (SSC) trajectories and outcomes of 29 P-MAC patients, of whom 26/29 (90%) had concurrent HIV infection, treated with guideline-based therapy (GBT) versus non-GBT regimens at a tertiary hospital in Gauteng province of South Africa between 2013 and 2017. We used ‘current value’ parameterisation of joint models with BERSs as longitudinal biomarkers for the composite outcomes of time-to-death or therapy failure.

**RESULTS:**

Overall, initial bacterial burden and BERS trajectories were heterogeneous. BERSs ranged from −0.034 to 0.013 log_10_ CFU/mL per day. BERSs were significantly steeper in the 18 (62%) censored survivors, with median (interquartile range) −0.011 (−0.021, −0.008), versus 0.002 (−0.008, 0.003) in 11 (38%) non-survivors or therapy failure. In multivariate analyses, one log_10_ CFU/mL/day decline was associated with an 88% reduction in the hazard for death or therapy failure (95% confidence intervals, 0.04, 0.34).

**CONCLUSION:**

BERSs from sputum are potential P-MAC treatment response biomarkers, which should be evaluated further in larger prospective studies of people with and without HIV.

The lack of quantitative and reproducible treatment response biomarkers is slowing, or even stalling, the development of novel regimens for pulmonary *Mycobacterium avium* complex (P-MAC) disease.^1,2^ Serial sputum culture (SSC) examinations are instrumental in the development of anti-TB drugs, particularly in early bactericidal activity (EBA) studies, where bacterial elimination rate slopes (BERSs) are used to rank the potency of drugs and combination regimens.^3–5^ However, the role of SSC as pharmacodynamic (PD) endpoints in P-MAC EBA studies is unclear.^6,7^ Specifically, P-MAC disease trajectories are highly heterogeneous, and a major challenge lies in defining therapeutic cure or identifying clinical endpoints that fully represent relapse-free sputum culture conversion (SCC).^8–10^ Providers often adjust drug doses and therapy combinations as well as patients’ prognosis intuitively based on clinical response, SCC, and radiological examinations. However, these qualitative measures do not inform therapy duration and are subject to bias. In addition, MAC organisms are ubiquitous in the environment, and disease recurrence due to relapse or reinfection occurs in up to 54% of treated patients, even among those with documented SCC.^11^ This makes SCC an inefficient PD endpoint since multiple negative cultures are required to confirm relapse-free culture conversion.^8,10,12^

Traditionally, MAC SSC responses, therapy duration, and patients’ survival outcomes are analysed separately, using mixed-effects models for longitudinal outcomes and survival models, such as Cox proportional hazard, for time-to-event analyses.^13^ However, SSC trajectories are often nonlinear and exhibit properties that violate standard statistical assumptions, complicating parameter estimations.^14^ For instance, SSCs are considered endogenous biomarkers and are susceptible to contamination during laboratory processing. Moreover, they are measured with errors that potentially bias bacterial load estimates. Additionally, SSCs are time-variant, and missing samples typically cannot be ignored because they often signify meaningful clinical outcomes such as death, cure, or bacterial loads below quantification levels. Here, we investigated whether BERSs could serve as viable PD endpoint and reproducible treatment response biomarker in extended P-MAC EBA studies. All-cause mortality was selected as a definitive hard endpoint because it is widely accepted in clinical trials for biomarker validation.^15,16^

Previously, we reported an all-cause mortality rate of 17 per 100 person-year among 123 patients with non-TB mycobacteria (NTM) in South Africa, most (73%) of whom had localised P-MAC disease, with 78% concurrently living with HIV.^17,18^ The cohort was highly heterogeneous, and as expected, baseline CD4 cell counts emerged as the primary and only predictor of survival.^17,19^ In this study, we reanalysed MAC SSC data from the same cohort, focusing on the composite endpoint of death or therapy failure as a time-to-event among patients with mean ≥2 sputum samples per patients. We hypothesised that low initial bacterial loads and steeper BERSs will correlate with faster SCC and more favourable outcomes.

## METHODS

Participants were recruited between 1 January 2013 and 31 December 2017, at Charlotte Maxeke Academic Johannesburg Hospital (CMJAH), a tertiary-level facility that serves Gauteng province and is the main teaching hospital for University of Witwatersrand, in South Africa.

### Inclusion and exclusion criteria

Patients with ≥2 sputum samples with speciation of MAC in the first sample met inclusion criteria. We excluded children, patients with positive blood cultures or tissue samples, and patients with samples considered laboratory contaminants. We extracted the following information: age, sex, weight, serial time to positive (TTP) values from the Mycobacteria Growth Inhibitor Tube (MGIT) system, HIV test results, CD4 counts, and therapy regimen received. Clinical outcomes, including death or therapy failure, were also documented. Therapy failure was obtained from provider notes or empirically defined as the change in the initially assigned guideline-based therapy (GBT) regimen to non-GBT. This therapy failure included therapy changes induced by adverse drug events. GBT was defined as receiving macrolide (clarithromycin or azithromycin) plus ethambutol with or without a rifamycin.^12^ Non-GBT regimens included TB drugs (first- or second-line) or other macrolide-free antimicrobials combinations given for different reasons, including poor drug tolerance. Drug susceptibility tests, including macrolide susceptibility tests, were not readily available. However, patients who did not receive any antimicrobial drugs were categorised under ‘watchful waiting’ or ‘untreated’, a management practice described in the 2007 American Thoracic Society (ATS)/Infectious Diseases Society of America (IDSA) treatment guidelines.^12^

### Mycobacterial cultures and estimation of bacterial load

The South African National Health Laboratory Services (NHLS) perform mycobacterial cultures and speciation of NTM of all clinical samples from CMJAH using the conventional assays recommended by the Clinical Laboratory Standards Institute.^12,20^ To compare BERS estimates and thresholds obtained from this study with those obtained from previous studies,^3,21,22^ we computed MAC log_10_ CFU per mL from TTP values using methods described by Magombedze et al.^3^ This method allows for TTP values obtained beyond the usual 2- to 3-week periods observed with EBA studies.^23^

### Statistical analysis

Given the small sample size of this pilot study, descriptive data were presented as both median and mean values, accompanied by their respective measures of spread: interquartile range (IQR) and standard deviations. To describe the evolution and BERS biomarker trajectory, we fitted different joint longitudinal and survival sub-models, allowing for shared random effects between them while guided by principles of parsimony and clinical theory.^24^ Survival was examined for the entire all-cause mortality follow-up, while the BERS was determined by the SSC available throughout the treatment duration for each patient. Models were fitted using maximum likelihood approach. Three separate models examined treatment, MAC species, and CD4 counts. The longitudinal biomarker sub-model was deliberately modelled as a linear mixed model, chosen a priori due to limited SSC data available for analysis. In contrast, the survival sub-model distribution was data-driven. The ‘current value’ parameterisation, which included MAC SSC trajectories as longitudinal biomarker, was used in the linear predictor of the survival sub-model. Several sub-models and distributions, including a flexible parametric sub-model, as well as Gompertz and exponential distributions, were examined in the survival sub-models before selecting the best model fit based on Akaike Information Criteria. An unstructured random effects variance–covariance matrix was applied, incorporating random intercept and slope to examine longitudinal trajectories. Additional models’ details are given in the Supplementary Data. In the final models, the GBT group was compared to the combined non-GBT and untreated ‘watchful waiting’ group. Sensitivity analyses were conducted to evaluate the effect of age, sex, and therapy duration. Missing CD4 count data were imputed for two HIV-negative patients (500 cells/cm^3^) and one HIV-positive patient (27 cells/cm^3^). The analyses were descriptive and hypotheses generation; therefore, multiple test corrections were not applied. R version 4, STATA version 16 (College Station, TX), and GraphPad version 9 (La Jolla, CA) software were used.

## RESULTS

**Figure 1.**
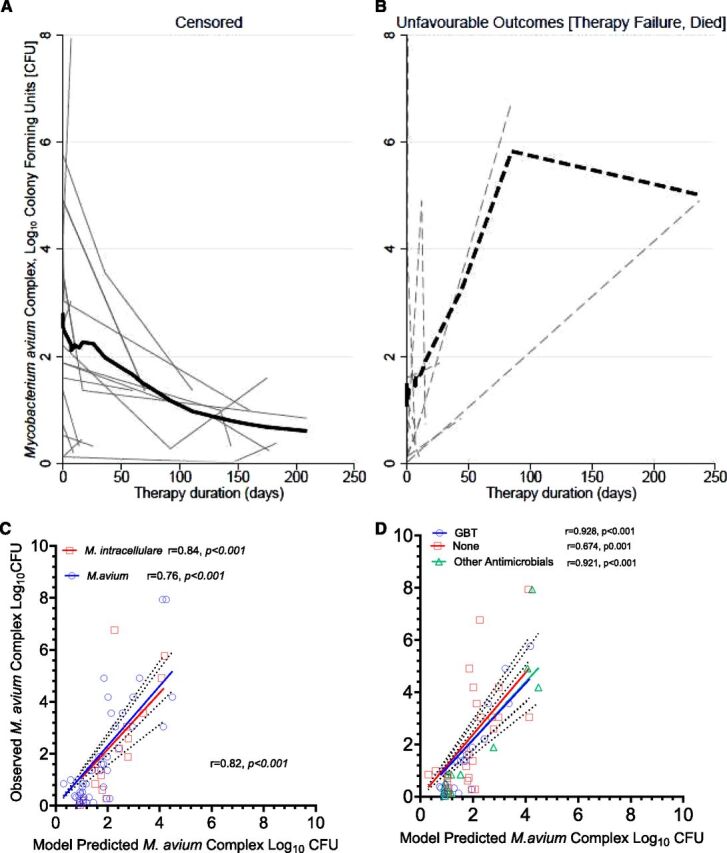
Observed and predicted *Mycobacterium avium* complex (MAC) burden. **A:** shows the individual trajectories of MAC serial sputum cultures (SSC) stratified by the composite event outcomes in censored or survivors and (**B**) in those who died or experienced therapy failure. Each faint grey line represents the trajectory of the quantitative SSC estimate in log_10_ colony-forming units (CFU) per mL over therapy duration and follow-up times (in days). The thick continuous line represents the estimated median bacterial elimination rate slope (BERS) in censored patients, while the interrupted represents median values in patients with unfavourable outcome events (death or therapy failure). **C:** shows the scatter plot of the observed MAC bacterial burden versus the model-predicted estimates stratified by *Mycobacterium avium* versus *Mycobacterium intracellulare* and **D:** shows by treatment regimens. As shown, the model underpredicted estimates in the untreated P-MAC patients.

Of the 34 patients who met the study inclusion criteria, five (15%) were excluded because of suspected contamination in three patients and disseminated MAC disease in two. Most patients, 26 (90%), had concurrent advanced HIV disease with acquired immunodeficiency syndrome, as evidenced by median and mean CD4 counts of 27 and 93 cells/mm^3^, respectively (Table 1). There was no significant difference in demographic and clinical characteristics between 6 (21%) participants with *M. intracellulare* versus 23 (79%) participants with *M. avium*. The exception was follow-up duration, which was significantly longer among participants with *M. intracellulare*, median (IQR) of 143 (85–177) versus 15 (7–70) days with *M. avium*, *P = 0.038*. We analysed 66 SSC, demonstrating heterogeneous trajectories of MAC bacterial load over time. These trajectories are illustrated in Figure 1A for participants with censored outcomes and Figure 1B for those with unfavourable (i.e., death or therapy failure) outcomes among the 29 (85% of the original recruited cohort).

**Table 1.** Clinical, demographic characteristics and outcomes of pulmonary *Mycobacterium avium* complex (P-MAC) patients enrolled in the study.

Characteristics	Value	All patients, N = 29 (%)	*M. avium*, n = 23	*M. intracellulare*, n = 6
Demographic				
Sex (%)	Female	11 (38%)	9 (39)	2 (33)
	Male	18 (62%)	14 (61)	4 (67)
Age, years	Median (IQR)	38 (30–45)	38 (30–51)	33 (30–42)
	Mean (SD)	39 (12)	41 (12)	33 (9)
Weight, kg	Median (IQR)	60 (46–76)	60 (45–68)	57 (50–76)
	Mean (SD)	61 (16)	61 (16)	60 (18)
Clinical				
HIV test	Negative/N/A	3 (10%)	2 (9)	1 (17)
	Positive	26 (90%)	21 (91)	5 (83)
CD4 counts (cells/mm^3^)	Median (IQR)	27 (12–111)	26 (5–72)	125 (23–384)
	Mean (SD)	93 (128)	65 (87)	200 (207)
Binary CD4 (cells/mm^3^)	≤50 cells/cm^3^	15 (52%)	13 (57%)	2 (33%)
	>50 cells/cm^3^	14 (48%)	10 (43%)	4 (67%)
Therapy	None	12 (41%)	10 (43%)	2 (33%)
	Non-GBT	7 (24%)	2 (22%)	2 (33%)
	GBT	10 (35%)	8 (35%)	2 (34%)
Outcomes				
Follow-up (days)	Median (IQR)	26 (9–111)	15 (7–70)	143 (85–177)
	Mean (SD)	65 (75)	48 (63)	132 (81)
Death or therapy failure	Died/failed	11 (38%)	9 (39%)	2 (33%)
Censored	Alive	9 (31%)	7 (30%)	2 (33%)
	Unknown	9 (31%)	7 (31%)	2 (34%)

GBT = guideline-based therapy; IQR = interquartile range; SD = standard deviation; CD4 = cluster of differentiation 4; N/A = data not available.

A strong correlation (rho [ρ]) was observed between the measured *M. avium* complex bacterial burden, expressed as log_10_ colony-forming units per millilitre (CFU/mL), and the model-derived estimates (Figure 1C and 1D). This correlation was consistent across most patient groups except for the untreated group. For the untreated group, the correlation coefficient was modest (ρ = 0.674, *P* < 0.001) compared to a significantly higher correlation for the GBT group (ρ = 0.928, *P* < 0.001), as shown in Figure 1D.

### BERS trajectories are heterogeneous, but the steeper ones predict favourable outcomes

**Figure 2.**
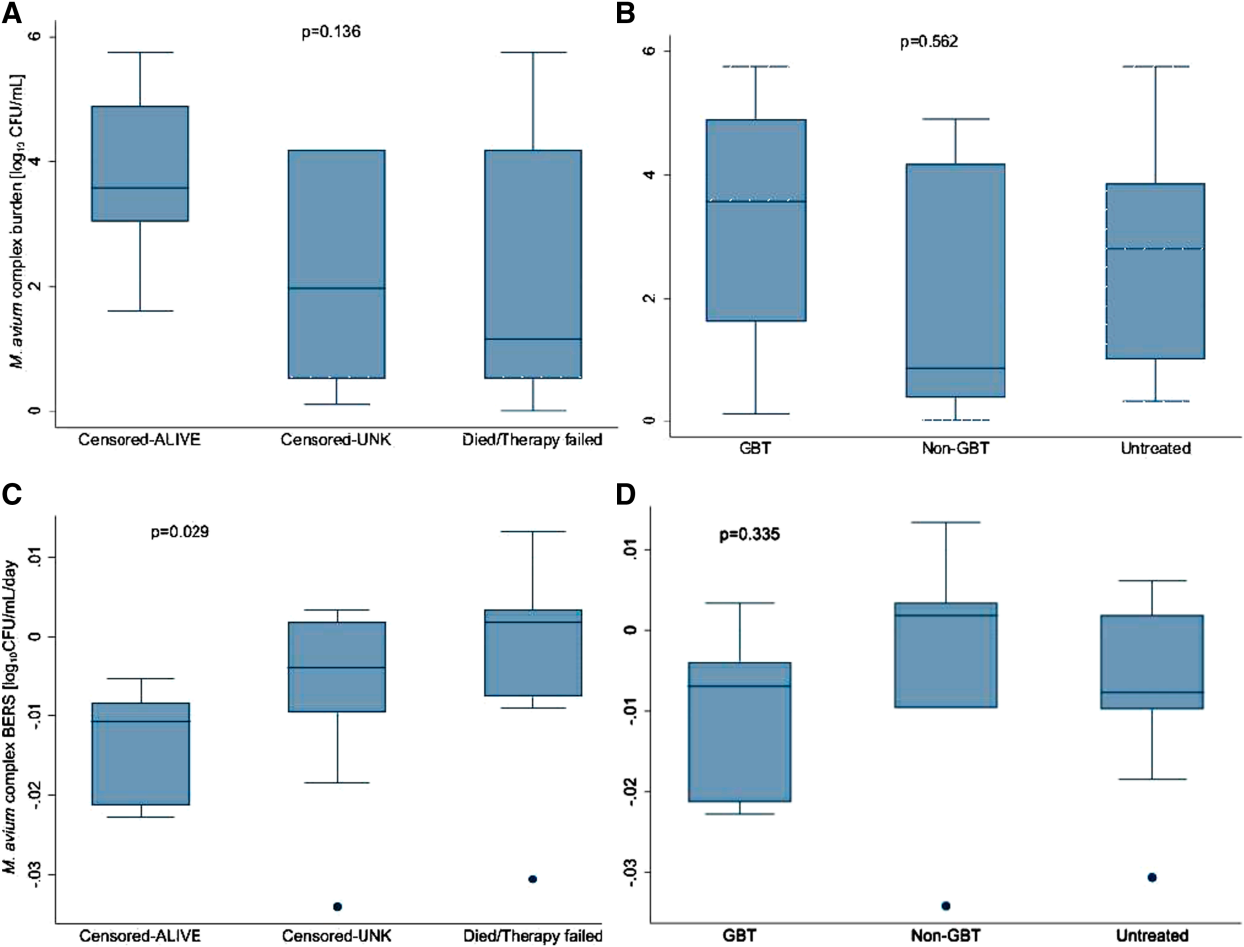
Comparison of the observed initial bacterial burden and bacterial elimination rate slope (BERS) estimates. **A:** shows the comparison of median values and overall distribution of the observed initial bacterial burden in log_10_ colony-forming units/mL (CFU/mL) by the composite outcomes and **B** shows treatment groups. The BERS estimates are shown in **C, D**, respectively. BERSs were significantly steeper in censored survivors, *P* = 0.029.

Using the linear mixed-effects models, we computed the initial MAC bacterial burden and the BERS. Overall, BERSs ranged from −0.034 to 0.013 log_10_ CFU/mL per day, with a mean of −0.007 and a median of −0.006 log_10_ CFU/mL per day. While initial MAC bacterial burden did not differ significantly between groups, BERSs were notably steeper in participants who survived compared to those who died or experienced therapy failure (*P* = 0.029). The median BERS (IQR) for survivors was −0.011 (IQR, −0.021 to −0.008) log_10_ CFU/mL/day, compared to 0.002 (IQR, −0.007 to 0.002) log_10_ CFU/mL/day for non-survivors or those with therapy failure. Together, these data suggest lower thresholds of −0.008 log_10_ CFU/mL/day for effective antimicrobial effect. When compared across different participant groups, BERSs exhibited significant between-individual variability, particularly across treatment and outcome groups (Figure 2; Supplementary Data Figure S1). For example, the percent coefficient of variation (% CV) was 90% in the GBT, 143% in the untreated, and 500% in the non-GBT groups. The high variability justified the use of linear mixed-effects models with both random intercepts and slopes. No significant differences in BERSs were observed across treatment regimens, MAC species, or CD4 cell count groups (Figure 2C and 2D and Supplementary Data Figure S1).

### Individual patients’ BERSs from sputum were independently associated with mortality outcomes

Table 2 presents the results from the joint longitudinal biomarker and survival models, which align with findings from the linear mixed-effects models, highlighting significant heterogeneity, particularly in the variability of the initial bacterial load and slope estimates. The ‘current biomarker value’ showed a strong and statistically significant association with the composite outcome risk which meant that joint models provided less biased estimates than separate analysis. The 2.11 (95% CI: 1.08, 3.14) estimate indicated an increased risk for poorer outcomes independent of GBT. One-unit decline in BERS (log_10_ CFU/mL per day) corresponded to an 88% reduction in the hazard ratio for death or therapy failure (95% CI: 0.04, 0.34).

**Table 2.** Examination of the joint impact of guideline-based therapy (GBT) on BERSs and the composite outcomes of death or therapy failure in pulmonary *Mycobacterium avium* complex patients.

Variable	Coefficient (95% confidence interval)	*P* value
Longitudinal biomarker (Log_10_ CFU)		
Time (days)	−0.011 (−0.014, −0.009)	<0.001
trt_01 (none/non-GBT vs. GBT)	0.175 (−0.697, 1.046)	0.694
Intercept (β_0_)	1.921 (0.657, 3.184)	0.003
Survival analysis (time to composite outcome: died/therapy-failed)		
Associative ‘current’ value	2.110 (1.082, 3.138)	<0.001
trt_01 (none/non-GBT vs. GBT)	−17.14 (−20.58, −13.70)	<0.001
_cons	9.471	
Random effects parameters		
sd (time)	0.008 (0.005, 0.013)	
sd (intercept, [β_0_])	0.780 (0.507, 1.198)	
sd (residuals)	1.707 (1.434, 2.031)	

BERS = bacterial elimination rate slope; CFU = colony-forming unit; sd = standard deviation; trt = treatment arm; cons = constant.

Adjusting treatment effects in both sub-models revealed a statistically significant impact of GBT on survival, albeit with a very large effect size compared to non-GBT. The lack of significant treatment effects on BERSs and the absence of differences among therapy regimens were unsurprising. This may be due to the diversity of drug combinations within the GBT and non-GBT categories, which likely contributed to high between-individual variability. Similarly, the influence of BERSs on survival remained unchanged in multivariate analyses that adjusted for MAC species and CD4 cell counts (Supplementary Data Table S1). Overall, these findings suggest that BERS ≤ −0.008 log_10_ CFU/mL per day is a more conservative threshold predictive of favourable outcomes, including survival, regardless of therapy regimen.

**Figure 3.**
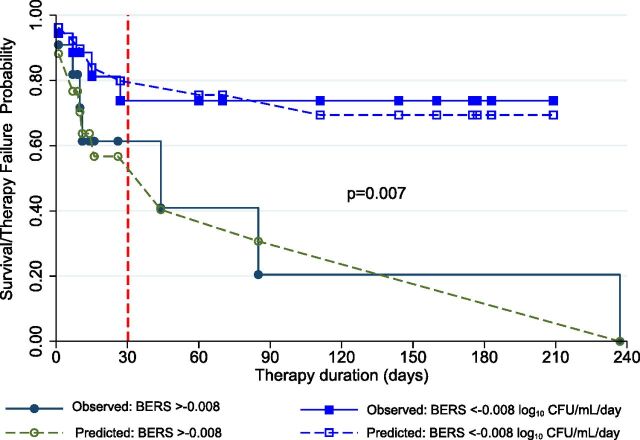
Comparison of shallow versus steep bacterial elimination rate slope (BERS) trajectories. We set −0.008 log_10_ CFU/mL per day as threshold to define steep (<−0.008) and compared to shallow (>−0.008). Dashed lines depict the ‘current value’ obtained from joint models. By day 30, the lines had diverged, with those in the shallow group experiencing higher fatalities. The 11 deaths or therapy failures led to an overall incidence rate of 5.81 per 1,000 person-day follow-up, which was estimated as 2.79 in those with steeper BERSs versus 15.22 with shallow BERSs per 1,000 person-day, respectively.

Finally, we compared the incidence rates of death or therapy failure between patients with BERSs below and above the threshold of −0.008 log_10_ CFU/mL per day. As shown in Figure 3, a divergence in outcomes became apparent by the end of the first month of therapy. P-MAC patients with BERSs > −0.008 had 5.46 times the incidence rate of death or therapy failure compared to those with steeper BERSs (≤−0.008 log_10_ CFU/mL per day) during therapy and follow-up (95% CI: 1.39, 25.64).

### Ethical statement

The University of the Witwatersrand Human Research Ethics Committee (Medical) approved the retrospective study (M180753).

## DISCUSSION

The primary objective of this exploratory analysis was to assess longitudinally measured quantitative MAC cultures obtained from sputum as potential biomarkers associated with survival endpoints during therapy. Baseline MAC bacterial load size, including semi-quantitative measures on culture and microscopy, and fibrocavitary disease observed in radiological examinations have previously been correlated with severe disease and worse outcomes.^25,26^ Our primary finding is that, across all models examined, the quantitative true value, or ‘current value’, of MAC sputum culture was predictive of the participant’s risk of death or therapy failure at that specific time point. An exponential survival model best fits our data, demonstrating that the entire participant-specific MAC SSC trajectory predicted the participants’ outcome within the 6-month observation period. This means that at any one point along therapy, an estimate of the ‘true quantitative bacterial burden’ predicts outcomes in that participant.

The modelling approach and findings contrast with the last-observation-carried-forward assumptions inherent in time-varying Cox proportional hazard models,^13,27^ often used when the longitudinal sputum culture biomarker model is analysed separately from the survival model. Those standard statistical models assume that SSC values remain constant between measurements – an assumption rarely met in real-world clinical settings, particularly when measurements are widely spaced. The joint models allowed for the inclusion of additional aspects of the biomarker (in this case, an estimated ‘current value’) to relate to the risk of an event. These data suggest that future efforts in P-MAC biomarker discovery, including the radiology space, are better served by utilising joint longitudinal and time-to-event models.^27^

Recent reports from a larger Dutch cohort of 49 patients, all treated with GBT, 57% of whom had cavitary MAC disease, corroborate our secondary findings. The authors suggested that an initial bacterial load >7 TTP days and a minimal slope increase threshold of 0.09 TTP days per day (approximately 0.109 log_10_ CFU/mL per day) within the first 3 months were predictive of sputum conversion outcomes.^25^ Therapy failure or survival outcomes were not examined. In this study, we identified a minimum slope threshold of −0.008 CFU/mL/day for P-MAC, which is an order of magnitude smaller than the one observed in the Dutch cohort but much closer to bacteriostasis observed with macrolide-based therapy in in vitro models. For MAC bacteraemia, a change of ≥1 log_10_ MAC CFU/mL from baseline is typically used as the threshold for partial mycobacteriologic response, therapy failure, or relapse.^28^ Similarly, others have suggested a threshold of 0.1 CFU/mL/day for pulmonary *M. tuberculosis*; however, baseline bacterial load and timing of the outcome measured influenced slope size.^3^ Although 3- and 6-months are the usual endpoints examined in clinical trials, our data suggest that BERSs can inform clinical decision-making as soon as 30 days into treatment. We hypothesise that differences in the P-MAC disease phenotypes, including the high prevalence of concurrent HIV infection rates in our cohort and variations in response to therapy due to different drug pharmacokinetic variability, may explain the smaller slope sizes observed. Regardless, the concept of a minimum quantitative PD response index to guide therapy could be transformative, particularly for NTM therapy, given the high rate of adverse drug events and rifampin narrow therapeutic window.

Finally, we did not identify significant differences in BERS estimates between therapy regimens, especially between the untreated or poorly treated groups and those on GBT. The explanation might be the heterogeneity of the GBT group, which increased the between-patient variability. Regardless, a steeper BERS observed in patients with >50 CD4 cell counts is consistent with the hypothesis that immune responses and anti-MAC drugs work via the BERS (Supplementary Data Figure S1).

However, caution must be exercised when interpreting these data due to small study sample size, limited longitudinal samples, and missing data. While the thresholds identified have been supported by prior hollow fibre data, larger prospective human cohorts are needed for further validation. For those future studies, more intensive sputum sampling during the first 3 weeks of therapy, when MAC drug tolerance typically develops, would likely be more informative than sampling at later time points near the end of therapy.^29^ In such cases, the area under the BERS curve could provide more precise associations between the longitudinal biomarker and time to death or therapy failure. Additionally, up to 30% of MAC isolates from clinical samples were not accounted for using culture-based methods, as they do not grow in standard culture media but remain viable and can persist to cause recurrent MAC disease in the human host.^30–33^ Finally, our findings might not be generalisable to P-MAC patients without concurrent HIV, as the pathogenesis and disease trajectories differ significantly in these populations. However, localised P-MAC disease with concurrent HIV remains poorly studied,^17,34,35^ and we believe these data will be valuable to the affected sub-Saharan patient population.

## CONCLUSION

We identified BERS thresholds, computed from SSC of P-MAC patients with concurrent HIV during the first 6 months of therapy, that associate with the composite outcomes of death or therapy failure. The findings need further investigations in prospective cohorts, including P-MAC patients without HIV.
